# Supplementary material for: Association between type 1 diabetes mellitus and educational attainment in childhood: a systematic review protocol
Source: BMJ Open. 2018 Aug 30;8(8):e021893. doi: 10.1136/bmjopen-2018-021893 (PMC6119409; doi:10.1136/bmjopen-2018-021893)
Supplement: Supplementary file 1 [file bmjopen-2018-021893supp001.pdf]

Appendix 1: OVID MEDLINE SEARCH STRATEGY

# ▲	Searches
1	exp Child/
2	exp Pediatrics/
3	exp Adolescent/
4	teen*.ti,ab.
5	child*.ti,ab.
6	adolescen*.ti,ab.
7	p?ediatric*.ti,ab.
8	juvenile*.ti,ab.
9	youth*.ti,ab.
10	(young adj3 (person* or people)).ti,ab.
11	minors.ti,ab.
12	or/1-11
13	exp Diabetes Mellitus, Type 1/
14	(type 1 diabetes or T1D or T1DM or diabet*).ti,ab.
15	(Insulin adj3 dependent).ti,ab.
16	13 or 14 or 15
17	(academic* adj3 (attain* or grade* or performance* or success* or status* or outcome* or result* or mark* or achiev* or score* or progress*)).ti,ab.
18	(educat* adj3 (attain* or grade* or performance* or success* or outcome* or result* or status* or mark* or achiev* or score* or progress*)).ti,ab.
19	(school* adj3 (attain* or grade* or performance* or success* or status* or outcome* or result* or mark* or achiev* or score* or progress*)).ti,ab.
20	exp Educational Status/
21	or/17-20
22	12 and 16 and 21
23	limit 22 to yr="2004 -Current"
