# Supplementary material for: Association between type 1 diabetes mellitus and educational attainment in childhood: a systematic review protocol
Source: BMJ Open. 2018 Aug 30;8(8):e021893. doi: 10.1136/bmjopen-2018-021893 (PMC6119409; doi:10.1136/bmjopen-2018-021893)
Supplement: Author's manuscript [file bmjopen-2018-021893.draft_revisions.pdf]

BMJ Open is committed to open peer review. As part of this commitment we make the peer review history of every article we publish publicly available.

When an article is published we post the peer reviewers' comments and the authors' responses online. We also post the versions of the paper that were used during peer review. These are the versions that the peer review comments apply to.

The versions of the paper that follow are the versions that were submitted during the peer review process. They are not the versions of record or the final published versions. They should not be cited or distributed as the published version of this manuscript.

BMJ Open is an open access journal and the full, final, typeset and author-corrected version of record of the manuscript is available on our site with no access controls, subscription charges or pay-per-view fees (http://bmjopen.bmj.com).

If you have any questions on BMJ Open's open peer review process please email info.bmjopen@bmj.com

BMJ Open

The Association between Type 1 Diabetes Mellitus and Educational Attainment in Childhood: A Systematic Review Protocol

Journal:	*BMJ Open*
Manuscript ID	bmjopen-2018-021893
Article Type:	Protocol
Date Submitted by the Author:	20-Feb-2018
Complete List of Authors:	Oakley, Natalie; Cardiff University School of Medicine, Division of Population Medicine<br>Hilliar, Mariann; University Hospital of Wales, Cardiff University Library<br>Mann, Mala; Cardiff University, University Library Services<br>Gregory, John<br>Dayan, Colin; Cardiff University School of Medicine<br>Tan, Jeanette; Cardiff University School of Medicine<br>Kneale, Dylan; UCL Institute of Education,<br>French, Robert; Cardiff University School of Medicine
Keywords:	DIABETES & ENDOCRINOLOGY, Educational attainment, Paediatric endocrinology < DIABETES & ENDOCRINOLOGY, Type 1 Diabetes Mellitus

SCHOLARONE™
Manuscripts

The Association between Type 1 Diabetes Mellitus and Educational Attainment in Childhood: A Systematic Review Protocol

Oakley N[1], Hilliar M[2], Mann M[3], Gregory JW[1], Dayan C[4], Tan J[4], Kneale D[5], French R*[4]
*Corresponding author: Dr Robert French. Email: frenchr3@cardiff.ac.uk
[1]Division of Population Medicine, Cardiff University School of Medicine, Neuadd Meirionnydd, University Hospital Wales, Cardiff, CF14 4YS.
[2]Cardiff University Library, Cochrane Building, University Hospital Wales, Heath Park, CF14 4YU.
[3]Specialist Unit for Review Evidence (SURE), Cardiff University School of Medicine, Neuadd Meirionnydd, University Hospital Wales, Cardiff, CF14 4YS.
[4]Cardiff University School of Medicine, University Hospital Wales, Heath Park, Cardiff, CF14 4YS.
[5]Social Science Research Unit (SSRU), University College London, 18 Woburn Street, London, WC1H 0NR

Abstract

Introduction
Global incidence of type 1 diabetes mellitus in childhood is rising, with the greatest increase noted in children under five years of age. Type 1 diabetes has the potential to significantly impact on children's school attainment. Quantifying this effect would be useful in assessing how much support and interventions should be focused on children with type 1 diabetes in school.

Methods and analysis
We will conduct a systematic review of all observational studies and randomised controlled trials including individuals both with and without a diagnosis of type 1 diabetes who have undertaken high stakes testing at the end of compulsory schooling when under 18 years of age. The search will cover both peer-reviewed and grey literature available from January 2004 to January 2018. The following seven databases will be searched: Ovid MEDLINE (R) (1946 to present), Ovid MEDLINE(R) Epub Ahead of Print, In-Process & Other Non-Indexed Citations, EMBASE (1947 to present), Web of Science, Education Resources Information Center (ERIC), British Education Index (BEI) and Cumulative Index to Nursing and Allied Health Literature (CINAHL). Study selection and data extraction will be performed independently by two reviewers with any disagreements resolved via a third reviewer. The quality and risk of bias in the observational studies included in this review will be assessed using the Newcastle-Ottawa Scale (NOS). We aim to conduct a meta-analysis and will assess heterogeneity between the included studies and potential for publication bias if sufficient studies.

Results and dissemination
Formal ethical approval is not required as individual patient data will not be collected. Results will be disseminated through peer-reviewed publication or conference presentations.

PROSPERO Registration number
CRD42017084078.

For peer review only

Strengths & Limitations of this study

- This systematic review will comprehensively evaluate available peer-reviewed and grey literature reporting the impact of type 1 diabetes on educational achievement in individuals undertaking high stakes standardised testing under age 18 at the end of compulsory schooling.
- Our findings will be reported using the recommended methods and checklist of the Preferred Reporting Items for Systematic Reviews and Meta-Analyses (PRISMA).
- Study selection and data extraction will be performed independently by two reviewers, with any disagreements resolved via a third reviewer.
- A potential limitation of this review may be varying quality and high heterogeneity amongst available studies.

Introduction

Background

Type 1 diabetes (T1DM), also known as insulin-dependent or juvenile diabetes, is an autoimmune disease which causes destruction of the insulin-producing beta cells in the pancreas, preventing the body from adequately regulating blood glucose levels.(1) The World Health Organisation has estimated that 347 million people worldwide have diabetes and it is believed that this number is likely to rise to 552 million by 2030.(2) Global incidence of type 1 diabetes mellitus in childhood is rising, with the greatest increase noted in children under five years of age.(3) Although in the population 10 per cent of people with diabetes have type 1,(4) it represents over 98% of childhood cases of diabetes.(5) Short term complications of diabetes can include hypoglycaemia, hyperglycaemia & ketoacidosis. Long term complications include heart disease, stroke, retinopathy, neuropathy and kidney disease.(6)

Rationale for Review

The health outcomes of type 1 diabetes in children are well documented, but the wider psycho-social impacts are less established and there is a lack of understanding of the effects on educational experience. These wider impacts are not only important in themselves, but also have the potential to have an effect on later life health outcomes through mechanisms such as employment, income and social status.

Many patients and their families express concerns about the potential negative impact that T1DM may have on a child's attainment at school. Hypoglycaemia, hyperglycaemia and diabetic ketoacidosis as well as psychological challenges and reduced attendance due to illness and hospital appointments are all factors which may theoretically result in poorer educational attainment for children with type 1 diabetes compared with their non-diabetic counterparts.(7-9) However, there is conflicting evidence as to the exact effect T1DM has on educational attainment and the real magnitude of this impact.(10)

Currently laws relating to managing children with chronic disease in school in the UK vary depending on specific country. In England, the Children and Families Act 2014 was introduced in September 2014 and imposed a statutory duty of schools to support children with medical conditions. The aim

For peer review only

of this was to ensure schools make additional arrangements for supporting these children, relating to both physical and mental wellbeing, allowing them to achieve their academic potential.(11)
As implied both in theory and in law, type 1 diabetes has the potential to significantly impact on children's school attainment. Therefore, assessing and analysing the current evidence to quantify this effect may be useful in assessing what and how much support and educational interventions should be focused on children with type 1 diabetes in school.

Objectives

The primary objective of this review is to assess and analyse the current literature available on whether type 1 diabetes has an impact on educational achievement in individuals undertaking high stakes standardised testing under 18 years of age at the end of compulsory schooling.
The secondary objectives include assessing the effect of type 1 diabetes on school attendance and educational attainment at other stages on the educational trajectory if reported.

Methods

We have used the Cochrane Handbook for Systematic Reviews of Interventions(12) to structure our methodological approach and we will report our findings using the recommended methods and checklist of the Preferred Reporting Items for Systematic Reviews and Meta-Analyses (PRISMA).(13) This protocol was created using the Preferred Reporting Items for Systematic Review and Meta-Analysis Protocols (PRISMA-P) guidelines.(14)

Eligibility criteria
The following criteria will be used to consider inclusion and exclusion of studies for this review.
Type of study:
We will include observational studies including prospective and retrospective cohort and case control studies (and randomised controlled trials if available). We will exclude case series, case reports and expert opinion/narrative reviews.
Population:
We will include studies including individuals who have undertaken high stakes testing at the end of compulsory schooling when under 18 years of age.
Intervention/Exposure:
Known diagnosis of type 1 diabetes before undertaking high stakes testing at the end of compulsory schooling.
Controls/Comparators:
No diagnosis of type 1 diabetes before undertaking high stakes testing at the end of compulsory schooling.
Outcome measures:
The primary outcome will be grades obtained at the end of compulsory schooling i.e. GCSE level or equivalent examinations.
Secondary outcomes may include school attendance and grades obtained at other stages on the educational trajectory if reported.
Time frame:
The 2015 NICE guidelines state that since 2004 there have been major changes in routine management of type 1 diabetes, aiming to achieve better glucose control to reduce long term

For peer review only

complications associated with the condition.(15) As a result, we will include studies published after the year 2004.

Setting:

Included studies will be secondary school based. Studies including outcomes from educational tests undertaken in clinical or other non-school settings will be excluded.

Search methods for identification of studies

We will search the following databases from 2004 to present, and will consider only studies published using the English language.

- Ovid MEDLINE (R) (1946 to present)
- Ovid MEDLINE(R) Epub Ahead of Print, In-Process & Other Non-Indexed Citations
- EMBASE (1947 to present)
- Web of Science
- Education Resources Information Center (ERIC)
- British Education Index (BEI)
- Cumulative Index to Nursing and Allied Health Literature (CINAHL)

Comprehensive electronic literature search strategies will be used for each database. See Appendix 1 for the Ovid MEDLINE and EMBASE search strategy.

To identify additional papers, information on studies in progress, unpublished research or research reported in the grey literature will be identified through searching a range of relevant websites, including Diabetes.org.uk, and trial registers including Clinical Trials.gov. We will search Electronic Table of Contents (eTOC) of key journals for relevant studies that have been published within the last two years. We also plan to check review articles, reference lists and carry out citation tracking of included studies for any significant studies missed during the database search.

Selection of studies

To select studies for further assessment, they will be imported and organised into Eppi-Reviewer 4.0(16) and duplicates will be removed. Two independent reviewers (NO & RF) will screen the titles and abstracts of every record retrieved from the searches using the predetermined inclusion criteria using Eppi-Reviewer 4.0.(16) Records identified as potentially eligible on the basis of title and abstract will then be screened on full text according to set inclusion criteria. If there is any doubt or disagreement regarding study selection, there will be further discussion and, if required, involvement of a third reviewer (JG) to reach a consensus. Rationale for exclusion of studies at this stage will be documented. The remaining included studies will then undergo data extraction using a standardised pro-forma. A PRISMA flow diagram will be used to demonstrate the number of included and excluded studies.

Data collection

All included studies will undergo data extraction by 2 independent reviewers (NO & RF), using a standardised pro-forma. The pro-forma will be pilot tested initially to ensure consistency.
Data extracted from each study will include:

- Details of study e.g. first author, date of publication, country/region where study undertaken.
- Details of study methodology e.g. study design, sample size, number of cases and controls included, inclusion/exclusion criteria.

For peer review only

- Modelling strategy and covariates/confounders adjusted for e.g. age, gender, socio-economic group.
- Outcomes – as stated below.

Again, any disagreements will be discussed and a third reviewer (JG) will be consulted if required.

Outcomes and prioritization

Primary outcome:

The primary outcome will be grades obtained at the end of compulsory schooling i.e. GCSE level or equivalent examinations. In most cases we expect this to be a continuous measure assessing scores across a range of subjects. We anticipate there may be some cases where a binary measure is used, for example, pass/fail.

Secondary outcomes:

The secondary objectives may include school attendance and grades obtained at other stages on the educational trajectory if reported. Again, in most cases, we expect these to be continuous measures.

Missing data

For any questions about eligibility or data not obtained from the full paper review, the authors of the papers will be contacted if required. If after 6 weeks no clarification has been provided, the study will be included in the final analysis & discussion however will be identified as ideally requiring further information.

Assessment of risk of bias in included studies

The quality and risk of bias in the observational studies included in this review will be assessed using the Newcastle-Ottawa Scale (NOS) for assessing the quality of non-randomised studies in meta-analysis.(17) The Newcastle-Ottawa scale assesses cohort & case control studies based on three domains:

1) Selection of study groups
2) Comparability of study groups
3) Ascertainment of Exposure (Case-Control studies)/ Outcome (Cohort studies)

Each study can be awarded a maximum of one star for each numbered component within the selection and exposure sections and a maximum of two stars can be given for the comparability section, creating a maximum of 9 stars per study. The higher the number of stars, the better quality the study and the lower the risk of bias.

If any RCTs are identified for inclusion in this review, we will assess the quality and risk of bias using the Cochrane Risk of Bias Tool, as described in the Cochrane Handbook for Systematic Reviews of Interventions.(18) This tool assesses risk of bias using five main domains: selection bias, performance bias, reporting bias, detection bias and attrition bias. It allows categorisation of risk of bias using three main outcomes: High, Low or Unclear.

In our review, this assessment will be completed by two independent reviewers (NO & RF). Any disagreements that cannot be resolved during moderation will be discussed with a third reviewer (JG).

Data synthesis

We will aim to conduct a meta-analysis using a random-effects model.

The majority of the outcome data from included studies in our review is likely to be continuous, therefore the measure of effect will be analysed using standardised mean difference with 95%

For peer review only

confidence interval. Any dichotomous outcome data will be analysed using risk ratios or odds ratios, which will also be converted to standardised mean difference with the appropriate transformations. In order to not lose information we will convert measures into a common metric and will aim to undertake sensitivity analyses to look for systematic difference according to transformations. We will use the statistical software Eppi-Reviewer 4.0(16) for our meta-analysis.
If possible, a sensitivity analysis will be performed to explore the impact of decisions made during the calculation of effect sizes, the inclusion of different study designs, and the impact of risk of bias assessments.
If we are unable to analyse data using meta-analysis, we will conduct a narrative synthesis. In this case, we will narratively summarise and tabulate the results found during data extraction in order to identify patterns in study design and outcomes across the included studies.

Assessment of heterogeneity

We will assess heterogeneity between the included studies by visual assessment of forest plots (for any minimal overlap) and use of statistical tests including the Chi^2 test and the I^2 statistic. If there is evidence of statistical heterogeneity, we will attempt to explore the reasons for the heterogeneity by using subgroup analyses based on the following:

- Patient demographics e.g. age, gender
- Diabetes specific characteristics e.g. age at diagnosis, HbA1c

We will also consider a random-effects meta-regression.

Publication-bias

We will examine funnel plots and conduct tests (Egger's test) to assess the potential for publication bias where there are sufficient (>10) studies.

Quality of overall body of evidence

We will assess the quality of evidence for all outcomes using the Grading of Recommendations Assessment, Development and Evaluation (GRADE) system. Risk of bias, directness, precision, heterogeneity and publication bias will be assessed and quality of the evidence will then be judged as high, moderate, low or very low. Results will be presented in 'Summary of findings' tables as recommended by the Cochrane Handbook for Systematic Reviews of Interventions.(18)

For peer review only

Footnotes

Contributorship statement

RF is the review guarantor. The concept of the review was proposed by RF & JG and the protocol manuscript was drafted by NO and edited by RF, MM & JG. The search strategy was designed by MH, NO & RF with advice from MM. NO, MM, JT & RF contributed to the development of the study eligibility criteria and data extraction criteria. JG & CD provided expertise on type 1 diabetes. MM provided expertise on systematic review methodology. DK provided expertise on data extraction & meta-analysis. All authors read, edited and approved the final manuscript.

Funding

This research was supported in part by the MRC grant MR/N015428/1.

Competing interests

All authors have completed the ICMJE uniform disclosure form at www.icmje.org/coi_disclosure.pdf and declare: RF has received a grant from the Medical Research Council *MR/N015428/1* for his work as principal investigator of the project 'Investigating the inter-relationship between diabetes and children's educational achievement'. All authors have no conflict of interest to report; no financial relationships with any organisations that might have an interest in the submitted work in the previous three years; no other relationships or activities that could appear to have influenced the submitted work.

Provenance and peer review

Not commissioned; externally peer reviewed.

REFERENCES

1. Kahanovitz L, Sluss PM, Russell SJ. Type 1 Diabetes - A Clinical Perspective. *Point Care* 2017;16(1):37-40.
2. Umpierrez GE. Therapy for Diabetes Mellitus and Related Disorders. 6th ed. Umpierrez GE, editor. Canada: American Diabetes Association; 2014.
3. Matyka K. Type 1 and type 2 diabetes mellitus in children. In: Wass J, Stewart P, editors. Oxford Textbook of Endocrinology and Diabetes. 2nd ed. Oxford: Oxford University Press; 2011.
4. DiabetesUK. Diabetes: the basics London: DiabetesUK 2017 [Available from: www.diabetes.org.uk/Diabetes-the-basics] (Accessed 8th December 2017)
5. Patterson CC, Dahlquist GG, Gyürüs E, et al. EURODIAB Study Group. Incidence trends for childhood type 1 diabetes in Europe during 1989–2003 and predicted new cases 2005–20: a multicentre prospective registration study. *The Lancet* 2009;373(9680):2027-33.
6. Diabetes.co.uk. Type 1 Diabetes UK: Diabetes.co.uk 2017 [Available from: www.diabetes.co.uk/type1-diabetes.html.] (Accessed 10th December 2017)
7. Glaab LA, Brown R, Daneman D. School attendance in children with Type 1 diabetes. *Diabet Med* 2005;22(4):421-6.
8. Gaudieri PA, Chen R, Greer TF, et al. Cognitive function in children with type 1 diabetes: a meta-analysis. *Diabetes Care* 2008;31(9):1892-7.
9. Bernstein CM, Stockwell MS, Gallagher MP, et al. Mental health issues in adolescents and young adults with type 1 diabetes: prevalence and impact on glycemic control. *Clin Pediatr (Phila)* 2013;52(1):10-5.

For peer review only

10. Cooper MN, McNamara KA, de Klerk NH, et al. School performance in children with type 1 diabetes: a contemporary population-based study. *Pediatr Diabetes* 2016;17(2):101-11.
11. Diabetes UK. Diabetes in schools - legal information 2017 [Available from: https://www.diabetes.org.uk/guide-to-diabetes/your-child-and-diabetes/schools/diabetes-in-schools-legal-information] (Accessed 8th December 2017).
12. Higgins J, Green S. Cochrane Handbook for Systematic Reviews of Intervention. Version 5.1.0 [updated March 2011]. The Cochrane Collaboration 2011 [Available from: http://handbook-5-1.cochrane.org/] (Accessed 8th December 2017)
13. Moher D, Liberati A, Tetzlaff J, et al. The PRISMA Group. Preferred Reporting Items for Systematic Reviews and Meta-Analyses: The PRISMA Statement. *PLoS Med* 2009;6(7):e1000097.
14. Moher D, Shamseer L, Clarke M, et al. Preferred Reporting Items for Systematic Review and Meta-Analysis Protocols (PRISMA-P) 2015 statement. *Syst Rev* 2015;4(1):1.
15. National Institute of Clinical Excellence. NICE guideline [NG18] Diabetes (type 1 and type 2) in children and young people: diagnosis and management. NICE; 2015.
16. Thomas J, Brunton J, Graziosi S. EPPI-Reviewer 4: software for research synthesis. EPPI-Centre Software,. London: Social Science Research Unit, UCL Institute of Education,; 2010.
17. Wells G, Shea B, O'Connell D, et al. The Newcastle-Ottawa Scale (NOS) for assessing the quality of nonrandomised studies in meta-analyses. 2013 [Available from: http://www.ohri.ca/programs/clinical_epidemiology/oxford.asp] (Accessed 8th December 2017).
18. The Cochrane Collaboration. Cochrane Handbook for Systematic Reviews of Interventions Version 5.1.0 [updated March 2011]. 2011.

For peer review only

Appendix 1: OVID MEDLINE SEARCH STRATEGY

# ▲	Searches
1	exp Child/
2	exp Pediatrics/
3	exp Adolescent/
4	teen*.ti,ab.
5	child*.ti,ab.
6	adolescen*.ti,ab.
7	p?ediatric*.ti,ab.
8	juvenile*.ti,ab.
9	youth*.ti,ab.
10	(young adj3 (person* or people)).ti,ab.
11	minors.ti,ab.
12	or/1-11
13	exp Diabetes Mellitus, Type 1/
14	(type 1 diabetes or T1D or T1DM or diabet*).ti,ab.
15	(Insulin adj3 dependent).ti,ab.
16	13 or 14 or 15
17	(academic* adj3 (attain* or grade* or performance* or success* or status* or outcome* or result* or mark* or achiev* or score* or progress*)).ti,ab.
18	(educat* adj3 (attain* or grade* or performance* or success* or outcome* or result* or status* or mark* or achiev* or score* or progress*)).ti,ab.
19	(school* adj3 (attain* or grade* or performance* or success* or status* or outcome* or result* or mark* or achiev* or score* or progress*)).ti,ab.
20	exp Educational Status/
21	or/17-20
22	12 and 16 and 21
23	limit 22 to yr="2004 -Current"

For peer review only

PRISMA-P (Preferred Reporting Items for Systematic review and Meta-Analysis Protocols) 2015 checklist: recommended items to address in a systematic review protocol*

Section and topic	Item No	Checklist item	Page
**ADMINISTRATIVE INFORMATION**			
Title:			
Identification	1a	Identify the report as a protocol of a systematic review	1
Update	1b	If the protocol is for an update of a previous systematic review, identify as such	N/A
Registration	2	If registered, provide the name of the registry (such as PROSPERO) and registration number	1
Authors:			
Contact	3a	Provide name, institutional affiliation, e-mail address of all protocol authors; provide physical mailing address of corresponding author	1
Contributions	3b	Describe contributions of protocol authors and identify the guarantor of the review	1
Amendments	4	If the protocol represents an amendment of a previously completed or published protocol, identify as such and list changes; otherwise, state plan for documenting important protocol amendments	N/A
Support:			
Sources	5a	Indicate sources of financial or other support for the review	1
Sponsor	5b	Provide name for the review funder and/or sponsor	1
Role of sponsor or funder	5c	Describe roles of funder(s), sponsor(s), and/or institution(s), if any, in developing the protocol	1
**INTRODUCTION**			
Rationale	6	Describe the rationale for the review in the context of what is already known	1-3
Objectives	7	Provide an explicit statement of the question(s) the review will address with reference to participants, interventions, comparators, and outcomes (PICO)	3, 4
**METHODS**			
Eligibility criteria	8	Specify the study characteristics (such as PICO, study design, setting, time frame) and report characteristics (such as years considered, language, publication status) to be used as criteria for eligibility for the review	4
Information sources	9	Describe all intended information sources (such as electronic databases, contact with study authors, trial registers or other grey literature sources) with planned dates of coverage	4, 5

For peer review only

Search strategy	10	Present draft of search strategy to be used for at least one electronic database, including planned limits, such that it could be repeated	7, 8
Study records:			
Data management	11a	Describe the mechanism(s) that will be used to manage records and data throughout the review	5, 6
Selection process	11b	State the process that will be used for selecting studies (such as two independent reviewers) through each phase of the review (that is, screening, eligibility and inclusion in meta-analysis)	5
Data collection process	11c	Describe planned method of extracting data from reports (such as piloting forms, done independently, in duplicate), any processes for obtaining and confirming data from investigators	5
Data items	12	List and define all variables for which data will be sought (such as PICO items, funding sources), any pre-planned data assumptions and simplifications	5
Outcomes and prioritization	13	List and define all outcomes for which data will be sought, including prioritization of main and additional outcomes, with rationale	5, 6
Risk of bias in individual studies	14	Describe anticipated methods for assessing risk of bias of individual studies, including whether this will be done at the outcome or study level, or both; state how this information will be used in data synthesis	6, 7
Data synthesis	15a	Describe criteria under which study data will be quantitatively synthesised	5-7
	15b	If data are appropriate for quantitative synthesis, describe planned summary measures, methods of handling data and methods of combining data from studies, including any planned exploration of consistency (such as I^2, Kendall's τ)	6, 7
	15c	Describe any proposed additional analyses (such as sensitivity or subgroup analyses, meta-regression)	6, 7
	15d	If quantitative synthesis is not appropriate, describe the type of summary planned	6
Meta-bias(es)	16	Specify any planned assessment of meta-bias(es) (such as publication bias across studies, selective reporting within studies)	7
Confidence in cumulative evidence	17	Describe how the strength of the body of evidence will be assessed (such as GRADE)	7

*** It is strongly recommended that this checklist be read in conjunction with the PRISMA-P Explanation and Elaboration (cite when available) for important clarification on the items. Amendments to a review protocol should be tracked and dated. The copyright for PRISMA-P (including checklist) is held by the PRISMA-P Group and is distributed under a Creative Commons Attribution Licence 4.0.**

From: Shamseer L, Moher D, Clarke M, Ghersi D, Liberati A, Petticrew M, Shekelle P, Stewart L, PRISMA-P Group. Preferred reporting items for systematic review and meta-analysis protocols (PRISMA-P) 2015: elaboration and explanation. BMJ. 2015 Jan 2;349(jan02 1):g7647.

For peer review only

BMJ Open

The Association between Type 1 Diabetes Mellitus and Educational Attainment in Childhood: A Systematic Review Protocol

Journal:	*BMJ Open*
Manuscript ID	bmjopen-2018-021893.R1
Article Type:	Protocol
Date Submitted by the Author:	05-Jul-2018
Complete List of Authors:	Oakley, Natalie; Cardiff University School of Medicine, Division of Population Medicine<br>Kneale, Dylan; UCL Institute of Education,<br>Mann, Mala; Cardiff University, University Library Services<br>Hilliar, Mariann; University Hospital of Wales, Cardiff University Library<br>Tan, Jeanette; Cardiff University School of Medicine<br>Dayan, Colin; Cardiff University School of Medicine<br>Gregory, John<br>French, Robert; Cardiff University School of Medicine
**Primary Subject Heading**:	Diabetes and endocrinology
Secondary Subject Heading:	Public health
Keywords:	DIABETES & ENDOCRINOLOGY, Educational attainment, Paediatric endocrinology < DIABETES & ENDOCRINOLOGY, Type 1 Diabetes Mellitus

SCHOLARONE™ Manuscripts

For peer review only

The Association between Type 1 Diabetes Mellitus and Educational Attainment in Childhood: A Systematic Review Protocol

Oakley N[1], Kneale D[2], Mann M[3], Hilliar M[4], Tan J[5], Dayan C[5], Gregory JW[1], French R*[5]
*Corresponding author: Dr Robert French. Email: frenchr3@cardiff.ac.uk
[1]Division of Population Medicine, Cardiff University School of Medicine, Neuadd Meirionnydd, University Hospital Wales, Cardiff, CF14 4YS.
[2]Social Science Research Unit (SSRU), University College London, 18 Woburn Street, London, WC1H 0NR
[3]Specialist Unit for Review Evidence (SURE), Cardiff University School of Medicine, Neuadd Meirionnydd, University Hospital Wales, Cardiff, CF14 4YS.
[4]Cardiff University Library, Cochrane Building, University Hospital Wales, Heath Park, CF14 4YU.
[5]Cardiff University School of Medicine, University Hospital Wales, Heath Park, Cardiff, CF14 4YS.

For peer review only

Abstract

Introduction
Type 1 diabetes has the potential to significantly impact on children's educational attainment. With the increase in incidence, quantifying this effect would be useful to assess how much additional support should be focused on children with type 1 diabetes in school.

Methods and analysis
We will conduct a systematic review of all observational studies and randomised controlled trials including individuals both with and without a diagnosis of type 1 diabetes who have undertaken high stakes testing at the end of compulsory schooling when under 18 years of age. The search will cover both peer-reviewed and grey literature available from January 2004 to January 2018. The following seven databases will be searched: Ovid MEDLINE (R) (1946 to present), Ovid MEDLINE(R) Epub Ahead of Print, In-Process & Other Non-Indexed Citations, Ovid EMBASE (1947 to present), Thomson Reuters Web of Science, EBSCO Education Resources Information Center (ERIC), EBSCO British Education Index (BEI) and EBSCO Cumulative Index to Nursing and Allied Health Literature (CINAHL). Study selection and data extraction will be performed independently by two reviewers with any disagreements resolved via a third reviewer. The quality and risk of bias in the observational studies included in this review will be assessed using the Newcastle-Ottawa Scale (NOS). We aim to conduct a meta-analysis and will assess heterogeneity between the included studies as well as potential for publication bias if sufficient (>10) studies are included.

Results and dissemination
Formal ethical approval is not required as individual patient data will not be collected. Results will be disseminated through peer-reviewed publication and conference presentations.

PROSPERO Registration number
CRD42017084078.

Strengths & Limitations of this study

• This systematic review will comprehensively evaluate available literature reporting the impact of type 1 diabetes on educational attainment in individuals undertaking high stakes standardised testing under age 18 at the end of compulsory schooling.
• Our findings will be reported using the recommended methods and checklist of the Preferred Reporting Items for Systematic Reviews and Meta-Analyses (PRISMA).
• Study selection and data extraction will be performed independently by two reviewers, with any disagreements resolved via a third reviewer.
• A potential limitation of this review may be varying quality and high heterogeneity amongst available studies.

For peer review only

Introduction

Background

Type 1 diabetes (T1DM), also known as insulin-dependent or juvenile diabetes, is an autoimmune disease which causes destruction of the insulin-producing beta cells in the pancreas, preventing the body from adequately regulating blood glucose levels. It can occur at any age but is most commonly diagnosed in childhood and adolescence.(1) According to the 2017 IDF Diabetes Atlas 8th edition, 451 million people aged 18-99 years worldwide are estimated to have diabetes, of which 7-12 percent are thought to have type 1 diabetes. The number of children diagnosed with type 1 diabetes is increasing annually, particularly in children under 15 years of age, with an estimated annual increase of approximately 3 percent. In 2017, there were an estimated 587,000 children under 15 years of age with type 1 diabetes worldwide, with an estimated 96,100 new cases every year.(2) In the UK, type 1 diabetes represents over 96 percent of childhood cases of diabetes.(3) Short term complications of diabetes can include hypoglycaemia, hyperglycaemia & ketoacidosis. Long term complications include heart disease, stroke, retinopathy, neuropathy and kidney disease.(4)

Rationale for Review

The health outcomes of type 1 diabetes in children are well documented, but the wider psycho-social impacts are less established and there is a lack of understanding of the effects on educational attainment.(5) These wider impacts are not only important in themselves, but also have the potential to have an effect on later life health outcomes through mechanisms such as employment, income and social status.

Many patients and their families express concerns about the potential negative impact that T1DM may have on a child's attendance at school,(6) and many report worries about schools' ability to support children with diabetes.(7) Hypoglycaemia, hyperglycaemia and diabetic ketoacidosis as well as psychological challenges and reduced attendance due to illness and hospital appointments are all factors which may result in poorer educational attainment for children with type 1 diabetes compared with their non-diabetic counterparts.(8-10) There is conflicting evidence as to the exact effect T1DM has on educational attainment and the real magnitude of this impact.(6)

Previous literature has focused on the effects of type 1 diabetes on cognitive functioning in children. In a meta-analysis in 2008, Gaudieri et al (9) found that paediatric type 1 diabetes was found to be associated with poorer performance in learning and memory skills as well as attention and executive function. They found that these lower cognitive scores were most pronounced with early-onset

diabetes. In a further meta-analysis published in 2009 by Naguib et al,(11) type 1 diabetes in childhood was found to be associated with mild cognitive impairments and mildly reduced overall intellectual functioning. In 2004, Desrocher et al (12) published a review of the neurocognitive outcomes in children with type 1 diabetes. They reported a range of deficits associated with type 1 diabetes with most significant effects found to be related to age of disease onset, hypoglycaemia, duration of effects and hyperglycaemia around puberty. More recently in a meta-analysis in 2018, He et al (13) found that glycaemic extremes associated with type 1 diabetes in childhood were associated with cognitive dysfunction, characterised by lowered intelligence, reduced attention and slower psychomotor speed. These findings from previous studies suggest a detrimental impact of type 1 diabetes in childhood on cognitive function, however there is less evidence whether this adversely impacts educational attainment in the form of results of high stakes examinations.

Each of the four home nations within the UK have made a commitment to support children & young people with medical conditions in school, including type 1 diabetes. Legislation varies across the home nations but all highlight the importance of support for children and young people with additional learning needs.(14) Under the Equality Act 2010, (15) all schools in England, Scotland and Wales have a duty to make reasonable adjustments to ensure that children and young people with a disability (including type 1 diabetes) are not discriminated against or put at a significant disadvantage to their peers. In England, the Children and Families Act 2014 (16) was introduced in September 2014. In January 2018, the National Assembly for Wales voted in favour of a new Additional Learning Needs and Education Tribunal Act (Wales) (17). In Scotland, there are a number of pieces of legislation regarding the rights of children with diabetes, in particular the Education (Additional Support for Learning) Act 2004 (Scotland) (18). Finally, in Northern Ireland, the Department of Education and Department of Health, Social Services and Public Safety published joint guidance entitled 'Supporting pupils with Medication Needs 2008'.(19)

As implied both in theory and in law, type 1 diabetes has the potential to significantly impact on children's educational attainment. Therefore, assessing and analysing the current evidence to quantify this effect may be useful in assessing what and how much support and educational interventions should be focused on children with type 1 diabetes in school.

Objectives

The primary objective of this review is to assess and analyse the current literature available on whether type 1 diabetes has an impact on educational attainment in individuals undertaking high stakes standardised testing under 18 years of age at the end of compulsory schooling.
The secondary objectives include assessing the effect of type 1 diabetes on school attendance and educational attainment at other stages on the educational trajectory if reported.

Methods

We have used the Cochrane Handbook for Systematic Reviews of Interventions (20) to structure our methodological approach and we will report our findings using the recommended methods and checklist of the Preferred Reporting Items for Systematic Reviews and Meta-Analyses (PRISMA).(21) This protocol was created using the Preferred Reporting Items for Systematic Review and Meta-Analysis Protocols (PRISMA-P) guidelines.(22) This protocol is registered with PROSPERO

For peer review only

(International Prospective Register of Systematic Reviews)(23) at the NHS Centre for Reviews and Dissemination (CRD) at the University of York. [Registration number: CRD42017084078].

Eligibility criteria

The following criteria will be used to consider inclusion and exclusion of studies for this review.

Type of study:

We will include observational studies including prospective and retrospective cohort and case control studies (and randomised controlled trials if available). We will exclude case series, case reports and expert opinion/narrative reviews.

Population:

We will include studies including individuals who have undertaken high stakes testing at the end of compulsory schooling when under 18 years of age.

Intervention/Exposure:

Known diagnosis of type 1 diabetes before undertaking high stakes testing at the end of compulsory schooling.

Controls/Comparators:

No diagnosis of type 1 diabetes before undertaking high stakes testing at the end of compulsory schooling. We will include studies using controls which allow estimates of an interpretable effect size, for example matched controls or population controls. We will record the type of control in data extraction and consider the implications in the review.

Outcome measures:

The primary outcome will be grades obtained in high stakes testing at the end of compulsory schooling.

Secondary outcomes may include school attendance and grades obtained at other stages on the educational trajectory if reported.

Time frame:

The 2015 NICE guidelines state that since 2004 there have been major changes in routine management of type 1 diabetes, aiming to achieve better glucose control to reduce long term complications associated with the condition.(24) We will therefore include studies published after the year 2004 in order to comprehensively evaluate the most up-to-date available peer-reviewed and grey literature. The effect on educational attainment associated specifically with these treatment changes from 2004 may only become apparent at a later stage and therefore only seen in more recent or future studies. As a result, while it is likely that many qualifying studies will use cohorts receiving treatment prior to this year, we will record this as part of our data extraction and consider this as part of the review comparison.

Setting:

Included studies will be secondary school based. Studies including outcomes from educational tests undertaken in clinical or other non-school settings will be excluded.

Search methods for identification of studies

We will search the following databases from January 2004 to January 2018, and will consider only studies published using the English language.

- Ovid MEDLINE (R) (1946 to present)
- Ovid MEDLINE(R) Epub Ahead of Print, In-Process & Other Non-Indexed Citations
- Ovid EMBASE (1947 to present)
- Thomson Reuters Web of Science
- EBSCO Education Resources Information Center (ERIC)

For peer review only

- EBSCO British Education Index (BEI)
- EBSCO Cumulative Index to Nursing and Allied Health Literature (CINAHL)

Comprehensive electronic literature search strategies will be used for each database. See Appendix 1 for the Ovid MEDLINE and Ovid EMBASE search strategy.

To identify additional papers, information on studies in progress, unpublished research or research reported in the grey literature will be identified through searching a range of relevant websites, including Diabetes.org.uk, and trial registers including Clinical Trials.gov. We will search Electronic Table of Contents (eTOC) of key journals for relevant studies that have been published within the last two years. We also plan to check review articles, reference lists and carry out citation tracking of included studies for any significant studies missed during the database search.

Selection of studies

To select studies for further assessment, they will be imported and organised into Eppi-Reviewer 4.0 (25) and duplicates will be removed. Two independent reviewers (NO & RF) will screen the titles and abstracts of every record retrieved from the searches using the predetermined inclusion criteria using Eppi-Reviewer 4.0.(25) Records identified as potentially eligible on the basis of title and abstract will then be screened on full text according to set inclusion criteria. If there is any doubt or disagreement regarding study selection, there will be further discussion and, if required, involvement of a third reviewer (JG) to reach a consensus. Rationale for exclusion of studies at this stage will be documented. The remaining included studies will then undergo data extraction using a standardised pro-forma. A PRISMA flow diagram will be used to demonstrate the number of included and excluded studies.

Data collection

All included studies will undergo data extraction by 2 independent reviewers (NO & RF), using a standardised pro-forma. The pro-forma will be pilot tested initially to ensure consistency.
Data extracted from each study will include:

- Details of study e.g. first author, date of publication, country/region where study undertaken.
- Details of study methodology e.g. study design, sample size, number of cases and controls included, inclusion/exclusion criteria, data linkage.
- Modelling strategy and covariates/confounders adjusted for e.g. age, gender, socio-economic group, age at diagnosis, duration of diabetes.
- Outcomes – as stated below.

Again, any disagreements will be discussed and a third reviewer (JG) will be consulted if required.

Outcomes and prioritization

Primary outcome:

The primary outcome will be grades obtained in high stakes testing at the end of compulsory schooling. In most cases we expect this to be a continuous measure assessing scores across a range of subjects. We anticipate there may be some cases where a binary measure is used, for example, achieving five GCSEs (grades A to C) is a commonly used benchmark in UK educational research.

Secondary outcomes:

The secondary objectives may include school attendance and grades obtained at other stages on the educational trajectory if reported. Again, in most cases, we expect these to be continuous measures.

For peer review only

Missing data

For any questions about eligibility or data not obtained from the full paper review, the authors of the papers will be contacted if required. If after 6 weeks no clarification has been provided, the study will be included in the final analysis & discussion however will be identified as ideally requiring further information.

Assessment of risk of bias in included studies

The quality and risk of bias in the observational studies included in this review will be assessed using the Newcastle-Ottawa Scale (NOS) for assessing the quality of non-randomised studies in meta-analysis.(26) The Newcastle-Ottawa scale assesses cohort & case control studies based on three domains:

1) Selection of study groups
2) Comparability of study groups
3) Ascertainment of Exposure (Case-Control studies)/ Outcome (Cohort studies)

Each study can be awarded a maximum of one star for each numbered component within the selection and exposure sections and a maximum of two stars can be given for the comparability section, creating a maximum of 9 stars per study. The higher the number of stars, the better quality the study and the lower the risk of bias.

If any RCTs are identified for inclusion in this review, we will assess the quality and risk of bias using the Cochrane Risk of Bias Tool, as described in the Cochrane Handbook for Systematic Reviews of Interventions.(27) This tool assesses risk of bias using five main domains: selection bias, performance bias, reporting bias, detection bias and attrition bias. It allows categorisation of risk of bias using three main outcomes: High, Low or Unclear.

We will also specifically analyse the linkage methodology used in all papers included, highlighting areas of potential bias which may impact on the overall quality of the studies.

In our review, this assessment will be completed by two independent reviewers (NO & RF). Any disagreements that cannot be resolved during moderation will be discussed with a third reviewer (JG).

Data synthesis

We will aim to conduct a meta-analysis using a random-effects model.

The majority of the outcome data from included studies in our review is likely to be continuous, therefore the measure of effect will be analysed using standardised mean difference with 95% confidence interval. Any dichotomous outcome data will be analysed using risk ratios or odds ratios, which will also be converted to standardised mean difference with the appropriate transformations. In order to not lose information we will convert measures into a common metric and will aim to undertake sensitivity analyses to look for systematic difference according to transformations. We will use the statistical software Eppi-Reviewer 4.0(25) for our meta-analysis.

If possible, a sensitivity analysis will be performed to explore the impact of decisions made during the calculation of effect sizes, the inclusion of different study designs, and the impact of risk of bias assessments.

If we are unable to analyse data using meta-analysis, we will conduct a narrative synthesis. In this case, we will narratively summarise and tabulate the results found during data extraction in order to identify patterns in study design and outcomes across the included studies.

Assessment of heterogeneity

For peer review only

We will assess heterogeneity between the included studies by visual assessment of forest plots (for any minimal overlap) and use of statistical tests including the Chi^2 test and the I^2 statistic. If there is evidence of statistical heterogeneity, we will attempt to explore the reasons for the heterogeneity by using subgroup analyses based on the following:

- Patient demographics e.g. age, gender
- Diabetes specific characteristics e.g. age at diagnosis, HbA1c

We will also consider a random-effects meta-regression.

Publication-bias

We will examine funnel plots and conduct tests (Egger's test) to assess the potential for publication bias where there are sufficient (>10) studies.

Quality of overall body of evidence

We will assess the quality of evidence for all outcomes using the Grading of Recommendations Assessment, Development and Evaluation (GRADE) system. Risk of bias, directness, precision, heterogeneity and publication bias will be assessed and quality of the evidence will then be judged as high, moderate, low or very low. Results will be presented in 'Summary of findings' tables as recommended by the Cochrane Handbook for Systematic Reviews of Interventions.(27)

Patient and Public Involvement

Patients were not involved in the development of this research question or systematic review protocol. Patients will not be involved in completion of the systematic review.

For peer review only

Footnotes

Contributorship statement

RF is the review guarantor. The concept of the review was proposed by RF & JG and the protocol manuscript was drafted by NO and edited by RF, MM & JG. The search strategy was designed by MH,

NO & RF with advice from MM. NO, MM, JT & RF contributed to the development of the study eligibility criteria and data extraction criteria. JG & CD provided expertise on type 1 diabetes. MM provided expertise on systematic review methodology. DK provided expertise on data extraction & meta-analysis. All authors read, edited and approved the final manuscript.

Funding
This research was supported in part by the MRC grant MR/N015428/1.

Competing interests
All authors have completed the ICMJE uniform disclosure form at www.icmje.org/coi_disclosure.pdf *and declare: RF has received a grant from the Medical Research Council MR/N015428/1 for his work as principal investigator* of the project 'Investigating the inter-relationship between diabetes and children's educational achievement'. All authors have no conflict of interest to report; *no financial relationships with any organisations that might have an interest in the submitted work in the previous three years; no other relationships or activities that could appear to have influenced the submitted work.*

Provenance and peer review
Not commissioned; externally peer reviewed.

REFERENCES

1. Krzewska A, Ben-Skowronek I. Effect of associated autoimmune diseases on type 1 diabetes mellitus incidence and metabolic control in children and adolescents. *BioMed Research International*. 2016; 2016: 6219730.
2. International Diabetes Federation. *IDF Diabetes Atlas, 8th edn.* **Brussels, Belgium: International Diabetes Federation, 2017.**
3. Royal College of Paediatric and Child Health. National Paediatric Diabetes Audit 2013-14. Report 1: Care Processes and Outcomes. Royal College of Paediatrics and Child Health; London (UK): Revised Sept 2015
4. Diabetes.co.uk (2017). Type 1 Diabetes [Available from: www.diabetes.co.uk/type1-diabetes.html] (Accessed 12th June 2018)
5. Persson S, Dahlquist G, Gerdtham UG and Steen Carlsson K. Impact of childhood-onset type 1 diabetes on schooling: a population-based register study. *Diabetologia* 2013; **56**: 1254-1262.
6. Cooper MN et al. School performance in children with type 1 diabetes: a contemporary population-based study. Pediatric Diabetes 2016; **17**: 101-111.
7. Streisand R, Monaghan M. Young Children with Type 1 Diabetes: Challenges, Research, and Future Directions. *Current diabetes reports*. 2014;14(9):520. doi:10.1007/s11892-014-0520-2.
8. Glaab LA, Brown R, Daneman D. School attendance in children with Type 1 diabetes. *Diabet Med* 2005;22(4):421-6.
9. Gaudieri PA, Chen R, Greer TF, et al. Cognitive function in children with type 1 diabetes: a meta-analysis. *Diabetes Care* 2008;31(9):1892-7.
10. Bernstein CM, Stockwell MS, Gallagher MP, et al. Mental health issues in adolescents and young adults with type 1 diabetes: prevalence and impact on glycemic control. *Clin Pediatr (Phila)* 2013;52(1):10-5.
11. Naguib et al. Neuro-cognitive performance in children with type 1 diabetes – a meta-analysis. *J Pediatr Psychol.* 2009; 34(3): 271-82.

For peer review only

12. Desrocher M, Rovet J. Neurocogntive correlates of type 1 diabetes mellitus in childhood. *Child Neuropsychol* 2004; 10(1): 36-52.
13. He J et al. Glycaemic extremes are related to cognitive dysfunction in children with type 1 diabetes: a met-analysis. J Diabetes Investig. 2018. doi: 10.1111/jdi.12840. [Epub ahead of print]
14. Diabetes UK (2017). Diabetes in schools - legal information. [Available from: https://www.diabetes.org.uk/guide-to-diabetes/your-child-and-diabetes/schools/diabetes-in-schools-legal-information] (Accessed 12th June 2018).
15. Equality Act 2010 (England, Scotland, Wales). [Available from http://www.legislation.gov.uk/ukpga/2010/15/pdfs/ukpga_20100015_en.pdf] (Accessed 12th June 2018)
16. Children and Families Act 2014 (England). [Available from http://www.legislation.gov.uk/ukpga/2014/6/pdfs/ukpga_20140006_en.pdf] (Accessed 12th June 2018)
17. Additional Learning Needs and Education Tribunal Act 2018 (Wales). [Available from http://www.legislation.gov.uk/anaw/2018/2/pdfs/anaw_20180002_en.pdf] (Accessed 12th June 2018)
18. Education (Additional Support for Learning) Act 2004 (Scotland). [Available from https://www.legislation.gov.uk/asp/2004/4/pdfs/asp_20040004_en.pdf] (Accessed 12th June 2018)
19. Department of Education and Department of Health, Social Services and Public Safety. Supporting pupils with Medication Needs 2008. [Available from: https://www.education-ni.gov.uk/sites/default/files/publications/de/supporting-pupils-with-medical-needs.pdf] (Accessed 12th June 2018)
20. Higgins J, Green S. Cochrane Handbook for Systematic Reviews of Intervention. Version 5.1.0 [updated March 2011]. The Cochrane Collaboration 2011 [Available from: http://handbook-5-1.cochrane.org/] (Accessed 8th December 2017)
21. Moher D, Liberati A, Tetzlaff J, et al. The PRISMA Group. Preferred Reporting Items for Systematic Reviews and Meta-Analyses: The PRISMA Statement. *PLoS Med* 2009;6(7):e1000097.
22. Moher D, Shamseer L, Clarke M, et al. Preferred Reporting Items for Systematic Review and Meta-Analysis Protocols (PRISMA-P) 2015 statement. *Syst Rev* 2015;4(1):1.
23. *PROSPERO: International Prospective Register of Systematic Reviews. 2012. NHS National Institute of Health Research. [Available from https://www.crd.york.ac.uk/PROSPERO/]* (Accessed 12th June 2018)
24. National Institute of Clinical Excellence. NICE guideline [NG18] Diabetes (type 1 and type 2) in children and young people: diagnosis and management. NICE; 2015.
25. Thomas J, Brunton J, Graziosi S. EPPI-Reviewer 4: software for research synthesis. EPPI-Centre Software,. London: Social Science Research Unit, UCL Institute of Education,; 2010.
26. Wells G, Shea B, O'Connell D, et al. The Newcastle-Ottawa Scale (NOS) for assessing the quality of nonrandomised studies in meta-analyses. 2013 [Available from: http://www.ohri.ca/programs/clinical_epidemiology/oxford.asp] (Accessed 8th December 2017).
27. The Cochrane Collaboration. Cochrane Handbook for Systematic Reviews of Interventions Version 5.1.0 [updated March 2011]. 2011.

For peer review only

Appendix 1: OVID MEDLINE SEARCH STRATEGY

# ▲	Searches
1	exp Child/
2	exp Pediatrics/
3	exp Adolescent/
4	teen*.ti,ab.
5	child*.ti,ab.
6	adolescen*.ti,ab.
7	p?ediatric*.ti,ab.
8	juvenile*.ti,ab.
9	youth*.ti,ab.
10	(young adj3 (person* or people)).ti,ab.
11	minors.ti,ab.
12	or/1-11
13	exp Diabetes Mellitus, Type 1/
14	(type 1 diabetes or T1D or T1DM or diabet*).ti,ab.
15	(Insulin adj3 dependent).ti,ab.
16	13 or 14 or 15
17	(academic* adj3 (attain* or grade* or performance* or success* or status* or outcome* or result* or mark* or achiev* or score* or progress*)).ti,ab.
18	(educat* adj3 (attain* or grade* or performance* or success* or outcome* or result* or status* or mark* or achiev* or score* or progress*)).ti,ab.
19	(school* adj3 (attain* or grade* or performance* or success* or status* or outcome* or result* or mark* or achiev* or score* or progress*)).ti,ab.
20	exp Educational Status/
21	or/17-20
22	12 and 16 and 21
23	limit 22 to yr="2004 -Current"

For peer review only

PRISMA-P (Preferred Reporting Items for Systematic review and Meta-Analysis Protocols) 2015 checklist: recommended items to address in a systematic review protocol*

Section and topic	Item No	Checklist item	Page
**ADMINISTRATIVE INFORMATION**			
Title:			
Identification	1a	Identify the report as a protocol of a systematic review	1
Update	1b	If the protocol is for an update of a previous systematic review, identify as such	N/A
Registration	2	If registered, provide the name of the registry (such as PROSPERO) and registration number	1
Authors:			
Contact	3a	Provide name, institutional affiliation, e-mail address of all protocol authors; provide physical mailing address of corresponding author	1
Contributions	3b	Describe contributions of protocol authors and identify the guarantor of the review	1
Amendments	4	If the protocol represents an amendment of a previously completed or published protocol, identify as such and list changes; otherwise, state plan for documenting important protocol amendments	N/A
Support:			
Sources	5a	Indicate sources of financial or other support for the review	1
Sponsor	5b	Provide name for the review funder and/or sponsor	1
Role of sponsor or funder	5c	Describe roles of funder(s), sponsor(s), and/or institution(s), if any, in developing the protocol	1
**INTRODUCTION**			
Rationale	6	Describe the rationale for the review in the context of what is already known	1-3
Objectives	7	Provide an explicit statement of the question(s) the review will address with reference to participants, interventions, comparators, and outcomes (PICO)	3, 4
**METHODS**			
Eligibility criteria	8	Specify the study characteristics (such as PICO, study design, setting, time frame) and report characteristics (such as years considered, language, publication status) to be used as criteria for eligibility for the review	4
Information sources	9	Describe all intended information sources (such as electronic databases, contact with study authors, trial registers or other grey literature sources) with planned dates of coverage	4, 5

For peer review only

Search strategy	10	Present draft of search strategy to be used for at least one electronic database, including planned limits, such that it could be repeated	7, 8
Study records:			
Data management	11a	Describe the mechanism(s) that will be used to manage records and data throughout the review	5, 6
Selection process	11b	State the process that will be used for selecting studies (such as two independent reviewers) through each phase of the review (that is, screening, eligibility and inclusion in meta-analysis)	5
Data collection process	11c	Describe planned method of extracting data from reports (such as piloting forms, done independently, in duplicate), any processes for obtaining and confirming data from investigators	5
Data items	12	List and define all variables for which data will be sought (such as PICO items, funding sources), any pre-planned data assumptions and simplifications	5
Outcomes and prioritization	13	List and define all outcomes for which data will be sought, including prioritization of main and additional outcomes, with rationale	5, 6
Risk of bias in individual studies	14	Describe anticipated methods for assessing risk of bias of individual studies, including whether this will be done at the outcome or study level, or both; state how this information will be used in data synthesis	6, 7
Data synthesis	15a	Describe criteria under which study data will be quantitatively synthesised	5-7
	15b	If data are appropriate for quantitative synthesis, describe planned summary measures, methods of handling data and methods of combining data from studies, including any planned exploration of consistency (such as I^2, Kendall's τ)	6, 7
	15c	Describe any proposed additional analyses (such as sensitivity or subgroup analyses, meta-regression)	6, 7
	15d	If quantitative synthesis is not appropriate, describe the type of summary planned	6
Meta-bias(es)	16	Specify any planned assessment of meta-bias(es) (such as publication bias across studies, selective reporting within studies)	7
Confidence in cumulative evidence	17	Describe how the strength of the body of evidence will be assessed (such as GRADE)	7

*** It is strongly recommended that this checklist be read in conjunction with the PRISMA-P Explanation and Elaboration (cite when available) for important clarification on the items. Amendments to a review protocol should be tracked and dated. The copyright for PRISMA-P (including checklist) is held by the PRISMA-P Group and is distributed under a Creative Commons Attribution Licence 4.0.**

From: Shamseer L, Moher D, Clarke M, Ghersi D, Liberati A, Petticrew M, Shekelle P, Stewart L, PRISMA-P Group. Preferred reporting items for systematic review and meta-analysis protocols (PRISMA-P) 2015: elaboration and explanation. BMJ. 2015 Jan 2;349(jan02 1):g7647.

For peer review only
